# One-arm safety intervention study on community case management of chest indrawing pneumonia in children in Nigeria – a study protocol

**DOI:** 10.1080/16549716.2020.1775368

**Published:** 2020-08-28

**Authors:** Helen Counihan, Ebenezer Baba, Olusola Oresanya, Olatunde Adesoro, Yahya Hamzat, Sarah Marks, Charlotte Ward, Patrick Gimba, Shamim Ahmad Qazi, Karin Källander

**Affiliations:** a Malaria Consortium, London, UK; b Malaria Consortium, Kampala, Uganda; c Malaria Consortium, Abuja, Nigeria; d Niger State Ministry of Health, Minna, Nigeria; e Department of Maternal, Newborn, Child and Adolescent Health, World Health Organisation, Geneva, Switzerland; f Department of Public Health Sciences, Karolinska Institutet, Stockholm, Sweden

**Keywords:** Under five, childhood pneumonia, oral amoxicillin, community health worker, CORPS, research design

## Abstract

Current recommendations within integrated community case management (iCCM) programmes advise community health workers (CHWs) to refer cases of chest indrawing pneumonia to health facilities for treatment, but many children die due to delays or non-compliance with referral advice. Recent revision of World Health Organization (WHO) pneumonia guidelines and integrated management of childhood illness chart booklet recommend oral amoxicillin for treatment of lower chest indrawing (LCI) pneumonia on an outpatient basis. However, these guidelines did not recommend its use by CHWs as part of iCCM, due to insufficient evidence regarding safety.

We present a protocol for a one-arm safety intervention study aimed at increasing access to treatment of pneumonia by training CHWs, locally referred to as Community Oriented Resource Persons (CORPs) in Nigeria. The primary objective was to assess if CORPs could safely and appropriately manage LCI pneumonia in 2–59 month old children, and refer children with danger signs. The primary outcomes were the proportion of children 2–59 months with LCI pneumonia who were managed appropriately by CORPs and the clinical treatment failure within 6 days of LCI pneumonia. Secondary outcomes included proportion of children with LCI followed up by CORPs on day 3; caregiver adherence to treatment for chest indrawing, acceptability and satisfaction of both CORP and caregivers on the mode of treatment, including caregiver adherence to treatment; and clinical relapse of pneumonia between day 7 to 14 among children whose signs of pneumonia disappeared by day 6. Approximately 308 children 2–59 months of age with LCI pneumonia would be needed for this safety intervention study.

## Background

Considerable progress has been made globally in the reduction of under-five mortality [1]. However, mortality remains high with 5.4 million deaths of children under 5 years old reported in 2017, of which 73% occurred across sub-Saharan Africa and Southeast Asia [1]. Pneumonia was a leading cause of these deaths, with an estimated 0.9 million pneumonia-related deaths in children aged 2–59 months in 2015; again over 75% of these deaths were in sub-Saharan Africa and Southeast Asia [2]. It is also a leading cause of death in Nigeria, accounting for 140,520 deaths in children under 5 years old in 2016 [3].

Mothers and other caregivers have an important role in early recognition of symptoms and in seeking immediate appropriate care for the sick child [4]. However, care seeking for pneumonia from an appropriate provider (accredited by the government) varies largely; from 27.2% in Ethiopia, 39.7% in Nigeria to 78.9% in Uganda [5]. Despite this, for children with signs of pneumonia, including chest indrawing for whom referral is indicated, many do not reach the referral facilities due to geographical, financial and socio-cultural barriers [6,7].

While recognising the important role of formal public and private health-care systems in service provision, current evidence suggests the need for a rapid expansion of access to low-cost, high-impact child health services at community-level to tackle common causes of child mortality [8–10]. Measures to improve care seeking for pneumonia by caregivers and appropriate training, support and supervision of community health workers (CHWs) can help reduce pneumonia-related mortality among children. These measures include enabling the CHWs to correctly assess, classify and manage fast breathing and chest indrawing pneumonia using oral antibiotic and counselling of caregivers on early care seeking [11,12]. A number of existing federal and state-level programmes in Nigeria provide community-level access to child health services, but often focus on single illnesses, lack effective linkages to formal, facility-based health services, and high coverage to have a sustained impact.

In April 2013, the Nigeria Federal Ministry of Health (FMoH) approved the national policy on integrated community case management (iCCM). The approach effectively extends the curative aspect of Integrated Management of Childhood Illness (IMCI) to the community level for the management of childhood malaria, pneumonia and diarrhoea. It is equity focused and is designed to provide prompt and effective case management of malaria, pneumonia and diarrhoea for children 2–59 months old in their communities. In addition, iCCM is expected to expand the scope, and improve effectiveness and integration with other community-based programmes such as health promotion. In Nigeria, iCCM services are provided by a designated community member known as a Community Orientated Resource Person (CORP), who are selected from and by their own communities to receive training as iCCM providers. They are supported through the provision of commodities and by supervising health facilities. Community sensitisation and mobilisation are also components of iCCM in Nigeria, to promote high levels of uptake by caregivers of children [13]. At the time of the study (2016–2018), the CORPs were providing iCCM services on a voluntary basis as per national guidelines.

The safety and efficacy of managing chest indrawing pneumonia with oral amoxicillin among children 2–59 months of age have been previously demonstrated at community level in Pakistan and Kenya [14–16]. These studies observed a lower need for referral or hospitalisation, and lower associated household and treatment costs [17]. It is postulated that community management of chest indrawing pneumonia would reduce transport, food and lost income costs for families; reduce pressure on already overburdened hospitals; and reduce risk of hospital-acquired infections. In 2012, the World Health Organization (WHO) revised its guidelines for the classification and treatment of childhood pneumonia for outpatient and inpatient hospital treatment [18]. WHO recommended oral amoxicillin for treatment of chest indrawing pneumonia on an outpatient basis at health facility level. It did not recommend its use by CHWs as part of iCCM, because there was still a lack of evidence about the safety of treating chest indrawing pneumonia in children at community level and the ability of CHWs to monitor their status to ascertain clinical deterioration and need for referral [19]. In 2016, data were unavailable from Africa on CHW management of chest indrawing pneumonia using oral amoxicillin in children 2–59 months of age, with the exception of the study in Kenya which was yet unpublished. To address this gap, a one-arm safety intervention study was conducted to assess the feasibility and capacity of CHWs in Nigeria to manage chest indrawing pneumonia in young children, instead of the current practice of referring such children to health facilities for management.

During the course of development of the study protocol including a customised study mobile app, and during data collection, considerable time was required for adaptation and refinement to adequately respond to the complexity of the topic. This was particularly in relation to ensuring the safety of the enrolled children and as a result, a unique study design and protocol were created which could serve as a prototype for similar studies. This paper presents the protocol for this study and will be used as a means to reference the underlying methodology in more detail within the result paper.

## Methods

### Study aim and objectives

The objective of this one-arm safety intervention study was to establish whether CHWs in Nigeria can safely manage chest indrawing pneumonia in 2–59 month old children with oral amoxicillin as part of an iCCM programme. A secondary objective was to assess the acceptability and satisfaction of such an approach among the CHWs as providers and the caregivers of children who were included.

### Study design

This one-arm safety intervention study was carried out in two local government areas (LGAs), Paikoro and Lapai in Niger state.

### Study site

**Figure 1.**
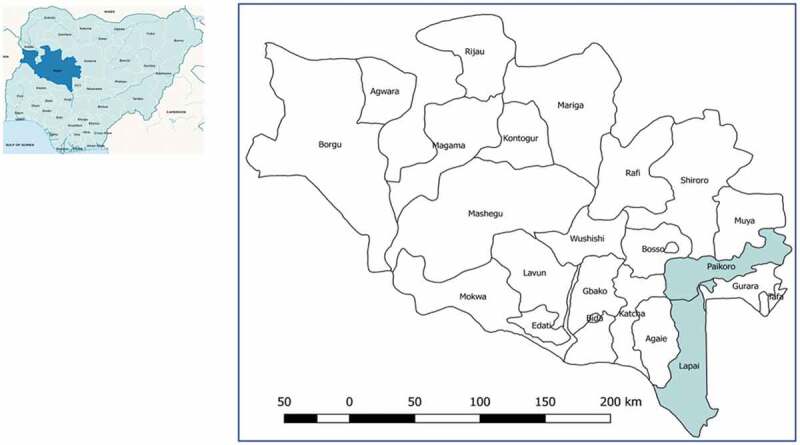
Nigeria map on left with Niger state marked, on right Niger state with two LGAs for chest indrawing pneumonia study.

Niger State, located in Nigeria’s North Central Zone, geographically the largest of the country’s states, has a population spread across rural areas with just 30% in urban areas. Children under 5 years make up 21% of the population, which is made up of Muslims, Christians and a small minority practising traditional beliefs. Under the WHO supported Rapid Access Expansion (RAcE) project, Malaria Consortium collaborated with the FMoH to establish and strengthen an iCCM programme in six LGAs in the state in 2013. The total population of these six LGAs was 1,245,939 of whom approximately 249,200 were children aged 2–59 months. The expected incidence of clinical pneumonia in this area in Nigeria was 0.28/child/year with an estimated 12% progressing to severe disease [20]. This study was conducted in two LGAs where the CORPs had been delivering iCCM services for at least 2 years before the start of the study – namely Lapai and Paikoro (Figure 1) with an approximate population of 164,000 and 222,200, respectively.

The selected LGAs had 356 CORPs (187 in Lapai and 169 in Paikoro) who had received a 6-day training on iCCM and had been equipped to provide these services for 2 years prior to the study. The objectives of the training were to build the capacity of the CORPs to detect and treat fast breathing pneumonia and diarrhoea and to diagnose and treat malaria with rapid diagnostic tests and artemisinin-based combination therapy, respectively. The training course was based on the WHO/UNICEF training programme ‘Caring for the sick child in the community’ and incorporated an integrated approach where the CORPs were trained to holistically assess the child and perform a differential diagnosis of their condition [21]. They were taught to recognise and refer cases with danger signs such as chest indrawing, give pre-referral treatment and refer as well as assess the nutritional and immunisation status of children under five.

### Study participants

A total of 356 CORPs across the two LGAs received additional training to be involved in the study. All children 2–59 months of age with possible chest indrawing but no danger signs (persistent vomiting, lethargic/unconscious, convulsions, not able to drink/breastfeed) were eligible for enrolment in the study. All sick infants younger than 2 months were referred to the supporting health facility. All children who were brought to CHWs in the two LGAs to receive care as part of iCCM services were potentially eligible to be included in the study. Caregivers of children who were classified as having chest indrawing pneumonia were given the option of having their child included in the study and thereby receiving treatment at home instead of being referred to a health facility as per the national iCCM guidelines.

### Intervention description

#### Sensitisation

Sensitisation meetings to introduce the study were organized at state and LGA levels. At state level, the main aim was to create awareness and increase the chances that research findings would inform implementation guidelines by programme implementers and policy-makers. At LGA level, consensus dialogues were organised for government staff, members of parliament and village chiefs. Support from community leaders was sought for disseminating messages to their respective communities, and to motivate households to seek care from the CORPs. Community mobilisation activities such as consultative meetings with community and religious leaders, as well as local announcements, were also organised at LGA and community levels. Through the RAcE project, there was already a strategy of community engagement which included community dialogues, which were community-led, with the CORP and community leader as facilitators. These meetings stimulated beneficial health-related decision making in the community. During the course of the study, the CORPs used this forum to share further the design and purpose of the study, answered community concerns and promoted community support and engagement.

#### Training

All trainings were designed by a capacity building specialist who directly trained the study team on how to train others on the study procedures. This team then conducted a 2-day training of 12 clinically trained research assistants (RA), who were a mix of community health extension workers and nurses, all of whom had also received training on iCCM. The RAs, in turn, conducted 3-day training courses for CORPS who were already trained in iCCM. These trainings included practical sessions for the CORPs to practise assessment of chest indrawing (using videos and visits to local hospital to see real cases), recruitment of children into the study including informed consent from caregivers and study protocol forms and steps. The study team also trained the supervisors from the reference health facilities that were supporting the CORPs in the LGAs where the study took place. As quality control measures, all trainings of the CORPs and their supervisors included pre- and post-tests, a training report completed by the trainers, and observations of trainings were conducted by the study team.

As a safety measure, RAs were also trained on a modified version of the WHO/UNICEF IMCI tool for their re-assessment of children to ensure no child was put at additional danger by being enrolled in the study. This training included the use of pulse oximetry as an extra precaution to ensure that enrolled children did not need referral for hypoxemia. The pulse oximeter given to the RAs was a Masimo iSpO2 Pulse Oximeter with micro USB connector for android devices with Masimo iSpO2 M_LNCS DCIP Pediatric Digit Sensor and Masimo LNCS YI Multisite Reusable Sensor with foam wrap for infants. The RAs’ main tasks were to confirm CORPs’ chest indrawing classification of children 2–59 months of age after recruitment and to collect data on primary and secondary outcomes during the follow-up visits. The RAs also assessed the child for any other illnesses, danger signs or other indications for referral. If detected, they gave advice and referred when required. Caregivers were also instructed to continue with the full antibiotic course as prescribed by the CORP to avoid the development of resistance, regardless of the result of the re-assessment by the RA or the health facility worker, even if they were diagnosed as not having chest indrawing pneumonia. If the child was referred to a health facility, to provide a history for the health staff receiving the child, the RA completed a form indicating clearly the results of their assessment, the reasons for referral and detailing the course of amoxicillin the child had been prescribed.

#### Identification of eligible children by CORPs

There were two stages for the identification and inclusion of eligible children into the study, as the RA had to confirm the CORP’s initial assessment before the child was fully included in the study. These stages are distinguished here by the use of the terms ‘recruitment’ for the CORP and ‘enrolment’ by the RA. The first stage with the CORP was the recruitment of the child into the study, which also included the giving of informed consent by the caregiver, and the allocation of a Unique Identifier Code (UIC) to the child by the CORP. The second stage was after the RA had completed their re-assessment of the child and they then enrolled the child into the study.

CORPs identified patients through passive surveillance and through periodic community outreach activities. Based on epidemiological data, it was expected that over a 12 month period of study data collection, each CORP would recruit 1–2 children with chest indrawing pneumonia. CORPs assessed all sick children presenting using the sick child assessment form and managed eligible children according to study protocol.

#### Consent and recruitment by CORPs on day 0

The CORPs recruited any eligible children on the consent of the caregiver. The CORPs gave literate caregivers the opportunity to read the consent form in full or read aloud to them if requested, explaining every aspect of the consent form to the caregiver, and answering any questions before the form was signed, or a thumbprint given, in triplicate by both the caregiver and the CORP. The CORP assigned a UIC to the child at this point.

**Figure 2.**
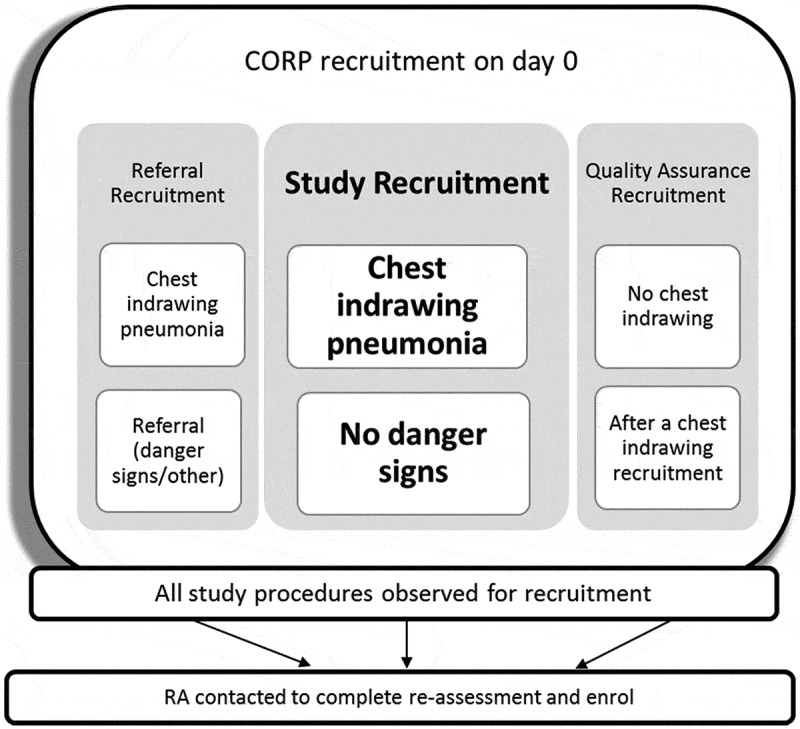
CORP recruitment: different types on day 0.

In total, the CORPs conducted three different types of recruitment on day 0 as shown in Figure 2 and detailed below.

- Study recruitment:

All children 2-59 months of age with chest indrawing but without other danger signs were considered eligible for study recruitment and consent obtained from the primary caregiver. The CORP gave the first dose of dispersible oral amoxicillin and observed the child for 30 minutes. If the child did not vomit s/he was considered recruited into the study. If the child vomited the first dose of amoxicillin, a repeat dose of oral amoxicillin was given and the child was excluded from the study if they vomited this also. The CORP provided the caregiver of the recruited child with dispersible amoxicillin tablets for 5 days according to the child’s age group (Table 1). The CORP provided any other treatments for malaria or diarrhoea if required and scheduled follow-up visits for day 0 (<12 hrs), 3, 6 and 15 with the caregiver of recruited child. The CORP then called the RA to notify them of the recruitment.

b. Referral recruitment:

**Table 1.** Dosing instructions for dispersible amoxicillin [19].

Age group	Weight	Dose recommendation	Total daily dose
2 to 11 months	4 to <10 kg	1 tab 2 times a day x 5 days	500 mg
12 to 35 months	10 to <14 kg	2 tabs 2 times a day x 5 days	1000 mg
36 to 59 months	14 to <19 kg	3 tabs 2 times a day x 5 days	1500 mg

All children 2–59 months with chest indrawing whose caregivers signed the consent form and who were subsequently referred were also followed up by the RA on day 0. The RA then followed up the referred child within 1 day after recruitment by the CORP, recording information regarding the child’s outcome in a tablet application. These children were included in the study as their data were necessary for the analysis of the primary outcome on the ability of CORPs to appropriately manage children with chest indrawing pneumonia.

c. Quality assurance recruitment:

As a quality assurance measure, once a CORP had enrolled a child with chest indrawing pneumonia, they were instructed to follow the study recruitment procedures for the next child they saw with fast breathing but without chest indrawing or danger signs. The purpose of this quality assurance recruitment was to assess their ability to correctly classify the absence of chest indrawing in children with suspected pneumonia. These children were provided standard iCCM care and treatment and were then re-assessed by the RA within 12 hours. No further data were collected on them.

#### Study enrolment by research assistants on day 0-1

The RA visited the recruited child within the following 12 hours to independently assess them using a tablet-based mobile data collection application, built on CommCare version 2.29.1 (Dimagi, Inc. Cambridge, MA, USA). The application also required the RAs to check if the child had received any other treatments in relation to their illness prior to presenting to the CORP, a factor included in the final analysis of treatment outcome. Eligible children were then included as enrolments by the RA on the application. If a child was found to have danger signs or hypoxaemia (SpO_2_ < 90%), or any other illness that was not treated by the CORP, the RA facilitated referral to the nearest health facility and the child was excluded from the study. If the RA did not detect chest indrawing and the caregiver indicated that there had been no history of chest indrawing, the RA assessed the CORP’s ability to identify chest indrawing pneumonia using videos of chest indrawing on the RA’s tablet. If the CORP passed this assessment, the child was then enrolled by the RA into the study.

#### Follow-up visit by CORPs on day 3

If the child was not brought by the caregiver on day 3 as per standard iCCM guidelines, the CORPs visited them at home. This was to ensure that there was no deterioration in the child’s condition and if necessary, to refer the child to a health facility. The CORP re-assessed the child, recorded the findings in a study form, and enquired if the caregiver provided the amoxicillin tablets according to the instructions given.

#### Follow-up visits by RAs on day 6 and day 15

On the 6th and 15th day after enrolment, the RA together with the CORP visited the enrolled children to re-assess them according to the IMCI protocol and interview the caregivers using the study data collection form on the tablet CommCare application. Questions included current health status, adherence to treatment (using pill count), care providers visited after visiting the CORP, alternative treatments given, and hospitalisation, if any reported by the caregiver. Clinical relapse of pneumonia (defined in Table 3) was determined based on the child’s clinical condition assessed on day 15.

**Table 2.** Follow-up visit schedule for children enrolled.

*Day*	0	0–1	3	6	15
Activity	**Screening and enrolment**	**Re-assessment**	**Re-assessment**	**Re-assessment**	**Re-assessment**
Person	CORP	RA	CORP	CORP & RA	RA
Outcomes		-CORP performance to classify & manage chest indrawing -Health status	-Health status	-Health status - Treatment failure -Treatment adherence	-Health status -Clinical relapse
Location	CORP	Household	CORP or Household	Household	Household

**Table 3.** Outline of study procedures including outcome assessment.

Activity	What	By whom	When	Where	How
Identification & recruitment of children with chest indrawing	Identification of children aged 2–59 months with chest indrawing and no other danger sign	CORP	Passively through provision of iCCM services	Home, community	iCCM assessment and identification of chest indrawing with no danger signs or other reason to refer
Provision of treatment	Oral amoxicillin (50–125 mg/kg) twice a day for 5 days	CORP	After consent obtained for recruitment in the study	Home, community	First dose given by CORP in presence of caregiver. Remainder of doses given by caregiver at home
Re-assessment of recruited child and full enrolment	Confirmation of chest indrawing pneumonia and absence of danger signs	RA	RA comes to child’s house within 12 hours of CORP recruitment (contacted by CORP)	Home	RA follows adapted IMCI tool to complete full assessment of child, including use of portable pulse oximeter; according to electronic study job aid application on tablet
Supervision and support	Supervision of adherence to study protocol, case confirmation, and follow up of recruited children including those then referred. Support of training, implementation, supply and logistics	RA for CORP, Study coordinator for RAs, Project manager for study coordinator. Support by study team and study sponsors. Assistance in development of tools by study team	Throughout the implementation	At home, community, health facility and hospitals	Accompanied visits, checking of registers, review of treatment cards, observation of administration of treatment, regular meetings and review of reports
Process evaluation and Monitoring	All aspects of implementation and its processes to understand if they are conducted as planned	Study team DSMB^a^ members Project manager and study coordinator (one visit)	On a weekly basis by the study team, throughout the implementation	At home, community, health facility and hospital	Review of recruitment forms, treatment cards, reports. Direct observation. Evaluate all processes, check whether processes and definitions as defined have been followed at each step, observe demand generation, check adequate supplies, storage facility and utilization practices Feedback shared with Government and corrective action taken
Outcome assessment	Clinical treatment failure of chest indrawing pneumonia by day 6 Defined as any of the following: Appearance of a danger sign^b^ Hypoxemia (Oxygen saturation ≤90%) Fever and chest indrawing on day 3 Fever or chest indrawing alone on day 6 Change of antibiotic by any health-care provider Death	Initially by RAs and then confirmed by study coordinator and study team	Periodically, throughout the implementation	At home, community, health facility and hospitals	Review of RA-completed electronic enrolment and follow-up forms; follow-up visits by study coordinator of referred children within a day for ascertaining outcome of referral, treatment advised and treatment taken
	Proportion of children 2–59 months classified with chest indrawing pneumonia who were managed appropriately by CORPs. Defined as Proportion of children enrolled with chest indrawing pneumonia and no other danger sign^b^, who received the correct age-specific dose. Proportion of children with chest indrawing pneumonia and referral sign who were given pre-referral treatment and referred to a health facility.	By RA	During their initial re-assessment of recruited child on day 0	Home, community, health facility	Re-assessment of child’s symptoms and verification of treatment given. If child had a referral sign, verification of any referral advice given by the CORP from the referral form
	Proportion of children classified with chest indrawing pneumonia who were followed up by the CORPs on day 3.	RA initially and by study team			From CORP recording form for day 3 visit
	Clinical relapse of pneumonia between day 7 to 14 among children whose signs of pneumonia disappeared by day 6. Defined as: Did not have chest indrawing on day 6 and developed any danger sign, chest indrawing, fever, and/or fast breathing.	RA	Day 15	Home, community	During re-assessment visit
	Adherence to treatment	RA and CORP	Day 6	Home	Pill count and questionnaire on administration of treatment
	Acceptability and satisfaction of caregivers	RA with additional training	Day 6	Home	Semi-structured interviews with caregivers
	Acceptability and satisfaction among CORPs	RA with additional training	Day 15	Community	Semi-structured interviews with CORPs

^a^Data and Safety Monitoring Board.

^b^Danger signs: unable to drink or breastfeed; convulsions; persistent vomiting after ingestion of food or drink; abnormally sleepy or difficult to wake; severe malnutrition.

The specific activities taking place for each of the follow-up visits can be found in Table 2. If on any of the follow-up days, enrolled children could not be found at home, the CORP/RA made two more attempts to follow up with the caregiver to trace the child. The study coordinator visited all the treatment failures for re-assessment of the child within 1 day of being notified by the RA, who would know of this through their own assessment or from the CORP almost immediately.

#### Quality assurance verification

During the study, to monitor the quality of the CORP’s classification of chest indrawing, a proportion of children who were brought to the CORP and classified as having fast breathing pneumonia were followed up by the RA and re-assessed following iCCM guidelines (Quality Assurance recruitments).

### Sample size

Sample size for a prevalence survey with finite population correction was calculated in STATA 13 (STATA Corp, College Station, TX, USA). Assuming that the proportion of treatment failure was 50% (using the most conservative estimate) a sample size of 196 children was needed to calculate the overall proportion of children with chest indrawing pneumonia who experienced treatment failure by day 6 with ±7% precision at 95% confidence level. Assuming a design effect of 1.3 to account for clustering of CORP performance in case more than one child is enrolled by one CORP [22], the total sample size required was 255 children; this was inflated to 308 to account for potential losses to follow-up including 10% non-response rate for caregivers and 10% erroneous enrolments.

### Outcomes

The primary outcomes measured were

- Proportion of children under 5 years classified with chest indrawing pneumonia who were managed appropriately by CORPs.
- Clinical treatment failure of chest indrawing pneumonia by day 6.

Secondary outcomes:

- Proportion of children under 5 years classified with chest indrawing who were followed up by the CORPs on day 3.
- Clinical relapse of pneumonia between day 7 to 14 among children under 5 years whose signs of pneumonia disappeared by day 6.
- Acceptability and satisfaction with community management of chest indrawing pneumonia among CORPs and caregivers.

Table 3 an outline of study procedures including outcome assessment.

### Data collection tools

To facilitate timely and complete data collection, as well as supporting quality assurance of CORP performance, an electronic data collection application was developed in CommCare for the study which was loaded onto electronic tablets (Tecno™ 7 C). This application was designed as a job aid for the RAs and also as a guided data entry process into electronic study forms and included different electronic forms for each stage of enrolment and follow-up of enrolled children. The application guided them through the WHO/UNICEF IMCI tool adapted for the study to verify the eligibility of the children recruited by the CORPs. This assessment included the use of a handheld pulse oximeter to check their oxygen saturation level and to confirm the medication and dosages prescribed. Each RA was provided with a locally available tablet on which the study CommCare app had been loaded, along with videos of chest indrawing, which were used as reference during assessment visits and for refresher training of the CORPs.

### Data management

A system for regular monitoring was established to ensure that study activities were implemented as per plan, using approved standard operating procedures. Each week, the Research Coordinator downloaded the data from the CommCare server and pasted it into an Excel-based data checker, which was used to highlight any duplicate entries, correct data entry mistakes reported by the RAs and to monitor progress on enrolment. The data checker was circulated to the study team on a weekly basis. The data manager regularly reviewed all data submissions for enrolled children, including study forms uploaded by RAs on day 0–1, 6 and 15, as well as follow-up forms submitted by CORPs on day 3. These submissions, which were anonymised from recruitment through the use of the UIC, were checked for completeness and accuracy. This UIC was also used to store the case data in a project database. While all data collection forms had inbuilt consistency and range checks, any additional discrepancies identified were raised during supervision visits.

### Qualitative component

The aim of the qualitative component of the study was to assess CORPs’ acceptability and caregiver acceptability and satisfaction with community-level management of chest indrawing pneumonia. This component was included in recognition of the importance of human behaviour and attitudes whenever introducing any new intervention, and this mix of qualitative and quantitative methodology is increasingly being adopted in clinical trials [23]. It involved conducting individual semi-structured interviews with CORPs on day 6 – this being their final day of follow-up of a child enrolled with chest indrawing. For the caregivers, the individual interviews were conducted with them on day 15 after the final day of follow-up by the RA for children recruited with chest indrawing pneumonia. For each group, there was a questionnaire developed which was used as the topic guide for the interview with different questions for each group. Four RAs with no previous experience in qualitative data collection were selected based on their previously observed levels of competence and because they were fluent both in the local language and in English. They were trained for 3 days by the study team on how to conduct and audio-record the interviews in the local language, and then translate and transcribe the data. This included role plays and field testing of the tools with CORPs and caregivers. Interviews were conducted until data saturation was reached in both groups.

### Data analysis

Main variables that were collected during the follow-up visits, using the study application on the RAs’ tablets included baseline characteristics of children and caregivers (children’s age and sex, caregivers’ age, sex, education, marital status, rural/urban residence). Clinical data for the children (both at recruitment into the study and at follow-up visits) included health status and symptoms present (fever, respiratory rate, chest indrawing) and danger signs. Treatment and care seeking history after initial presentation to CORPs including any visit to other providers and secondary treatment with antimicrobial medicines prescribed – which should have been recorded as a treatment failure. Data were also collected on adherence to the amoxicillin treatment schedule and any hospitalisation and mortality among the children.

The data were exported from the mobile data collection forms into Excel and then exported to STATA 13 for analysis. The primary analysis was conducted on the intention-to-treat population (including all children for whom the primary outcome was collected). Pearson’s Chi-squared test was used to test for association between categorical data accounting for clustering using the svy command in STATA 13. The baseline characteristics of the children and caregivers were summarised using descriptive statistics with means (SD) and medians (IQR). Proportions of the primary outcomes (CORPs’ performance, treatment failure) and secondary outcomes (adherence to follow-up, adherence to treatment, clinical relapse) were calculated and presented with 95% confidence intervals (CI).

The qualitative data from the transcriptions were analysed following a thematic analysis using ‘Framework Approach’ [24] using MAXQDA (VERBI Software, Berlin, Germany, 2016) to manage data coding, searching and retrieval. A sample of the transcriptions of recordings from local language into English was checked for accuracy in translation by the study team. Another sample of English translations was reviewed by the qualitative research specialist for data quality purposes (e.g. to check for sufficient probing and adherence to the topic guide). Summaries of each theme were reviewed and discussed by the study team before the final consolidation.

### Quality assurance

Quality assurance (QA) exercises were conducted immediately after the initial training, 1 month after training and again 6 months after training. An experienced iCCM trainer was used as the reference standard evaluator and assessed a group of study staff (CORPs and RAs). The CORPs were shown at least two different videos of children with or without chest indrawing and any misclassification was considered to be an error.

At the early stages of the study, a Data and Safety Monitoring Board (DSMB) was established to review periodic reports from the study, as well as compliance with the study protocol. The DSMB worked as an independent body comprised five experts, who served in their individual capacity to provide an assessment of the project and its outcomes.

## Discussion

This study had several strengths within its design which can allow it to serve as a model for evaluating the safety and acceptability of a new treatment intervention at community level, within the context of an already functioning iCCM programme. A key requirement was that no child should be put at any additional risk by being enrolled in the study, while at the same time, the procedure for delivering the intervention needed to be as close to real-life setting as possible. This was made possible by adding two components to the study design – the RAs who verified the CORPs’ assessments and by the use of an electronic data collection application which allowed data to be transmitted in near real-time. RAs were required to conduct verification visits within 12 hours of the child being seen by the CORP, and to conduct a comprehensive assessment based on IMCI guidelines, as would be expected if the child was seen in a health facility. Furthermore, the RAs were equipped with pulse oximeters to measure oxygen saturation so that any seriously hypoxaemic child (SpO_2_ < 90%) would be detected and immediately referred [25].

## Paper context

An evaluation was yet to be completed in sub-Saharan Africa of the safety and acceptability of community health workers, based in remote rural communities, to manage chest indrawing pneumonia in young children. This protocol is a unique contribution to the literature in presenting a methodology by which this evaluation can be achieved within a routine health delivery programme while avoiding children being put at any additional risk.
